# Application of CO_2_ Supercritical Fluid to Optimize the Solubility of Oxaprozin: Development of Novel Machine Learning Predictive Models

**DOI:** 10.3390/molecules27185762

**Published:** 2022-09-06

**Authors:** Saad M. Alshahrani, Ahmed Al Saqr, Munerah M. Alfadhel, Abdullah S. Alshetaili, Bjad K. Almutairy, Amal M. Alsubaiyel, Ali H. Almari, Jawaher Abdullah Alamoudi, Mohammed A. S. Abourehab

**Affiliations:** 1 Department of Pharmaceutics, College of Pharmacy, Prince Sattam Bin Abdulaziz University, P.O. Box 173, Al-Kharj 11942, Saudi Arabia; 2 Department of Pharmaceutics, College of Pharmacy, Qassim University, Buraidah 52571, Saudi Arabia; 3 Department of Pharmaceutics, College of Pharmacy, King Khalid University, Abha 62529, Saudi Arabia; 4 Department of Pharmaceutical Sciences, College of Pharmacy, Princess Nourah bint Abdulrahman University, Riyadh 145111, Saudi Arabia; 5 Department of Pharmaceutics, Faculty of Pharmacy, Umm Al-Qura University, Makkah 21955, Saudi Arabia; 6 Department of Pharmaceutics and Industrial Pharmacy, College of Pharmacy, Minia University, Minia 61519, Egypt

**Keywords:** optimization, solubility, mathematical modeling, green chemistry, machine learning

## Abstract

Over the last years, extensive motivation has emerged towards the application of supercritical carbon dioxide (SCCO_2_) for particle engineering. SCCO_2_ has great potential for application as a green and eco-friendly technique to reach small crystalline particles with narrow particle size distribution. In this paper, an artificial intelligence (AI) method has been used as an efficient and versatile tool to predict and consequently optimize the solubility of oxaprozin in SCCO_2_ systems. Three learning methods, including multi-layer perceptron (MLP), Kriging or Gaussian process regression (GPR), and k-nearest neighbors (KNN) are selected to make models on the tiny dataset. The dataset includes 32 data points with two input parameters (temperature and pressure) and one output (solubility). The optimized models were tested with standard metrics. MLP, GPR, and KNN have error rates of 2.079 × 10^−8^, 2.173 × 10^−9^, and 1.372 × 10^−8^, respectively, using MSE metrics. Additionally, in terms of R-squared, they have scores of 0.868, 0.997, and 0.999, respectively. The optimal inputs are the same as the maximum possible values and are paired with a solubility of 1.26 × 10^−3^ as an output.

## 1. Introduction

In the last years, disparate scientific investigations have been conducted on advanced targeted drug delivery systems owing to the need of pharmaceutical industries. Indeed, developing appropriate methodologies for particle engineering with the aim of controlling particle size is of great importance due to the drastic impact of this parameter on the drug delivery route [1,2,3].

A supercritical fluid (SCF) is identified as any fluid above critical pressure/temperature, where its density follows the behavior of liquids, but its viscosity and diffusivity follow a manner between liquid and gas. Moreover, SCFs possess a surface tension near zero. Considering their brilliant transport characteristics, SCFs have been of great interest for application in various industrial activities like extractions, chromatography, and particle engineering [4,5,6,7,8]. SCFs can be an appropriate option for poisonous and explosive light hydrocarbons and organic solvents [9,10,11,12,13]. Amongst various SCFs, it seems that carbon dioxide SCF (CO_2_SCF) can be considered the only commonly applied “green solvent” due to its very low flammability, inert nature, simplicity of utilization, and low threshold limit value (TLV). It is worth noting that the TLV amount of CO_2_SCF is significantly more eco-friendly and less poisonous than acetone (TLV = 750 ppm) or pentane (TLV = 600 ppm) [14].

Nowadays, the development of mathematical modeling and numerical simulations to compare the experimental (real) results with predicted ones is an important and efficient activity towards moving the quality-by-design (QbD) paradigm in the pharmaceutical industry [15,16,17]. Artificial intelligence (AI) is a novel and promising technique for developing predictive models in disparate industrial processes, such as membrane-based separation, crystallization, coating, and chemical reactions [18,19,20,21].

Machine learning (ML) methods are gradually replacing traditional computing methods in a variety of scientific disciplines. These problem-solving strategies include neural networks, ensemble models, and tree-based models. Machine learning models may now be used to study many difficulties by several initial properties and several final amounts. The correlation among initial amount and final values are derived by these methods [22,23,24]. In this work, three distinct methods including GPR, KNN, and MLP are selected to make models on the available dataset.

GPR has recently gotten much attention as a powerful statistical technique for data-driven modeling. GPR’s popularity stems partly from its theoretical connection to Bayesian nonparametric statistics, infinite neural networks, kernel approaches in machine learning, and spatial statistics [25,26].

The name “MLP” refers to a multi-layer perceptron-based neural network. MLPs are forward-feeding artificial neural networks. MLP has at least three levels: inputs, outputs, and hidden layers. The input layer nodes are not active; instead, the input layer nodes represent the data point. The input layer will have d nodes if the data point is presented by a d-dimensional vector [27,28].

The central idea of k-nearest neighbors (KNN) models is that they use the similarity of input data attributes to generate forecasts using other points that are most like the first. More specifically, it retains the entire training data during the testing phase [29,30].

## 2. Data Set

**Figure 1:**
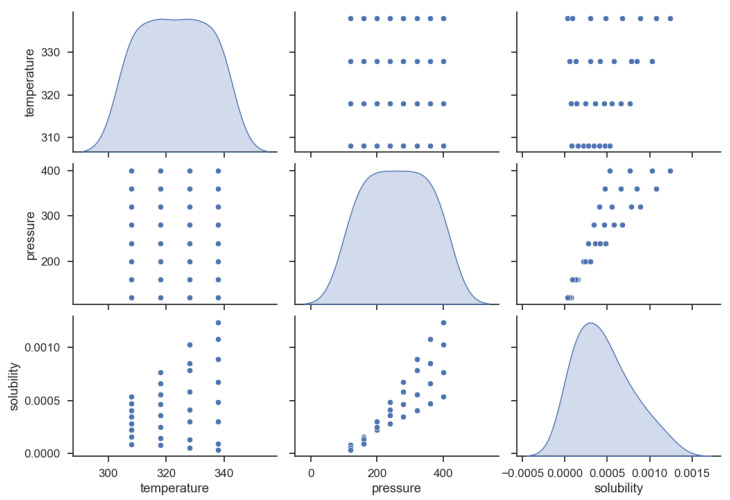
Pairwise Distribution of variables.

The used dataset of this study was taken from [31] which only has 32 data vectors. Each vector contains two input parameters (temperature and pressure) and one output (solubility). The dataset is shown in Table 1 and pairwise distribution of parameters is displayed in Figure 1.

## 3. Methodology

### 3.1. Gaussian Process Regression

Based on the Bayesian theory, it is possible to consider the GPR as a random process that employs the Gaussian processes to implement a nonparametric regression [32,33]. In this case, according to the Gaussian distribution, the probability distribution over function (*x*) for each input is determined as follows:

(1) $$f\left(x\right)\sim GPR\left(m\left(x\right),k\left(x,x^{\prime }\right)\right)$$

Here (*x*) and (*x*, *x*′) represent the mean and covariance functions, respectively. These functions are computed using the following equations:

(2) $$\{\begin{array}{c} m\left(x\right)=E\left(f\left(x\right)\right)\\ k\left(x,x^{\prime }\right)=E\left[\left(m\left(x\right)-f\left(x^{\prime }\right)\right)\left(m\left(x\right)-f\left(x^{\prime }\right)\right)\right] \end{array}$$

In which () denotes the expectation value. In practice, the value of (*x*) is usually considered equal to zero for simplifying the process of calculation. It should be noted that this assumption leads to erroneous results [32]. For describing the correlation degree between an expected target value of the training data set and the predicted target according to the resemblance of the respective inputs, the (*x*, *x*′) is also called the kernel function.

In a regression problem, the prior distribution of outputs *y* is defined as follows:

(3) $$y\sim N\left(0,k\left(x,x^{\prime }\right)+\sigma_{n}^{2}I_{n}\right)$$

where (), and *σ_n_* specify a normal distribution and the noise term, respectively. It is assumed that a similar Gaussian distribution exists between the testing subset *x*′ and training subset *x*. In this case, the forecast outputs *y*′ would track a joint prior distribution through the training output *y* as [34]:

(4) $$\left[\begin{array}{c} y\\ y^{\prime } \end{array}\right]\sim N\left(0,\left[\begin{array}{cc} k\left(x,x\right)+\sigma_{n}^{2}I_{n} & k\left(x,x^{\prime }\right)\\ k{\left(x,x^{\prime }\right)}^{T} & k\left(x^{\prime },x^{\prime }\right) \end{array}\right]\right)$$

Here *k*(*x*, *x*), *k*(*x*′, *x*′), and *k*(*x*, *x*′) denote the covariance matrices between input variables from the training set, testing set, and training-testing sets, correspondingly.

In the training process, with the help of the *n* points, some hyper-parameters *θ* present in the covariance function are optimized to warranty the application of GPR. Minimizing the negative log marginal likelihood *L*(*θ*) is a way to reach an optimized answer as [35]:

(5) $$\{\begin{array}{c} L\left(\theta \right)=\frac{1}{2}\log\left[det\lambda \left(\theta \right)\right]+\frac{1}{2}y^{T}\lambda^{-1}\left(\theta \right)y+\frac{n}{2}\log\left(2\pi \right)\\ \lambda \left(\theta \right)=k\left(\theta \right)+\sigma_{n}^{2}I_{n} \end{array}$$

As the optimized settings of the hyper parameters of GPR are determined, the forecast output *y*′ is calculated at dataset *x*′ by determining the related conditional distribution *p*(*y*′|*x*′, *x*, *y*) as:

(6) $$p(y^{\prime }|x^{\prime },x,y\sim N\left(y^{\prime }|\bar{y^{\prime }},cov\left(y^{\prime }\right)\right)$$

with:

(7) $$\{\begin{array}{c} \bar{y^{\prime }}=k{\left(x,x^{\prime }\right)}^{T}{\left[k\left(x,x\right)+\sigma_{n}^{2}I_{n}\right]}^{-1}y\\ cov\left(y^{\prime }\right)=k\left(x^{\prime },x^{\prime }\right)-k{\left(x,x^{\prime }\right)}^{T}{\left[k\left(x,x\right)+\sigma_{n}^{2}I_{n}\right]}^{-1}k\left(x,x^{\prime }\right) \end{array}$$

In which $\bar{y^{\prime }}$ represents the related mean values of the forecast. (*y*′) represents a variance matrix to determine the uncertainty range of these forecasts. These equations of GPR are explained in detail in [32].

### 3.2. Multilayer Perceptron Neural Networks

The feed-forward neural networks that include several latent layers are known as the multi-layer perceptron (MLP). One of the ways to develop the broadly employed MLP is the training rule of back propagation, which is rooted in the learning rule of error-correction (it is equivalent to traveling in the minus orientation of the immediate deviation according to the error function, that decreases the mistakes) [36,37].

The rule of back propagation includes two methods:

- First, the input vector is involved to the multilayer network, and its influences are transferred to the output levels over the hidden (middle) layers. Then, the final vector created on the latent class generates the genuine response of MLP.
- Next, in the backward path, the MLP settings are updated and regulated. The rule of error-correction will be followed in the implementation of this regulation. Furthermore, in the middle layers, weights of neurons are adjusted to reduce the difference between the neural network’s predicted results and its actual results [38,39].

When the ANN is developed, the data will be basically split into two periods of trial. No rules are available to minimize the size of training and test datasets [40,41].

In MLP, the procedure starts only with initial class and proceeds up to the nerve cells in the final class to yield some results. A hidden class is any layer that exists between these two layers (input and output). The activation functions, solver function, and quantity of hidden layers are hyperparameters in this algorithm that should be optimized. The output formulation for the MLP model with one hidden layer and a single output is as follows [42,43]:

(8) $$\tilde{y}=\delta_{2}\left(\overset{m}{\underset{i=1}{\sum }}\left(w_{i}^{\left(2\right)}\delta_{1}\left(X\right)\right)+b^{\left(2\right)}\right)X=\overset{n}{\underset{j=1}{\sum }}\left(x_{j}w_{xj}^{\left(1\right)}\right)+b^{\left(1\right)}$$

Here, $\tilde{y}$ represents the estimation vector of the MLP model, *m* indicates the data vector amount in the entire data set, n denotes initial amount details in the dataset, and **x***_j_* is *j^th^* feature vector **w**^(2)^ reflects the weights among the latent class and the final class, whereas **w**^(1)^ indicates the weights of initial attributes linked to the latent class. $\delta_{2}$ is the activation obligation for the final class [44]. In addition, the neurons’ activation function is $\delta_{1}$ in the latent class. **b**^(2)^ and **b**^(1)^ represent the bias vectors in the final class and all latent classes [45].

### 3.3. KNN

The KNN regression, starts to learn by contrasting the identified data points with the training dataset [30]. To explain this method, we assume $T=\left\{\left(x_{1},y_{1}\right),\ldots ,\;\left(x_{N},y_{N}\right)\right\}$ is the training data and $x_{i}=\left(x_{i1},x_{i2},\ldots ,x_{im}\right)$ indicates the *i^th^* data point with its *m* input features and the output is *y_i_*. *N* represents the quantity of data points. It must calculate the *d_i_* between a test instance *x* and every sample *x_i_* in *T* and sort the *d_i_* distance by its value for a test sample *x*. If *d_i_* rates in the *i^th^* position, the *d_i_* matching sample is referred to as the *i^th^* close to *NNi*(*x*), then the target is denoted as *y_i_* (*x*). Lastly, the estimation $\hat{y}$ of *x* denotes the average of the regression outputs of *k^th^* close to *x*, i.e., $\hat{y}=\frac{1}{k}\overset{k}{\underset{i=1}{\sum }}y_{i}\left(x\right)$. The KNN workflow regression algorithm is as follows [29,46]:

- Inputs: training input vectors $\left\{x_{i},y_{i}\right\},\;x_{i}$: input features, $y_{i}$: real–valued output, testing point *x* to predict
- Algorithm:
  ○ calculate distance $D\left(x,x_{i}\right)$ to every training example $x_{i}$
  ○ select *k^th^* nearest input vector $x_{i1}\ldots x_{ik}$ and their outputs $y_{i1}\ldots y_{ik}$
  ○ output:
    (9) $$\hat{y}=f\left(x\right)=\frac{1}{K}\overset{k}{\underset{j=1}{\sum }}y_{i_{j}}$$

## 4. Results

In this section, after examining the adjusted hyper-parameters stated in the previous section, the final models will be generated and compared with the criteria in this field to evaluate and analyze the results of the suggested models with the data. R^2^-score and MSE metrics are used in this study:

- For effective regression models, the R-square (R^2^) score is an actuarial metric. These graphs demonstrate a varies amount of percentage among related and non-aligned variables. It is critical to be able to quickly quantify the 0 to 100% difference among the related vary and the regression model.
- A mean squared error is one other standard metric for calculating the output of regression methods. MSE squares the points on the regression line. If the value’s negative sign is deleted and larger variances are given more weight, the squared value becomes significant. The lower the mean fault, the better match you will detect. The sooner, the best.

**Figure 2:**
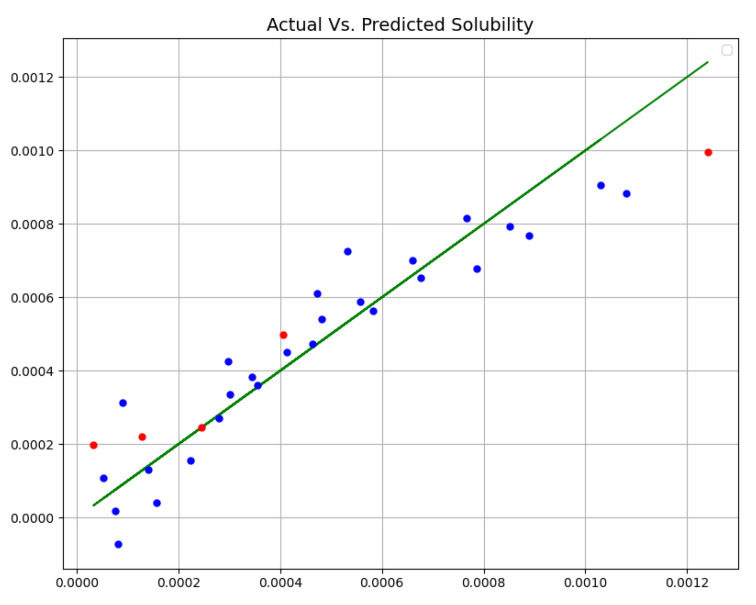
Actual Vs. Predicted Solubility (mole fraction) (MLP).

**Figure 3:**
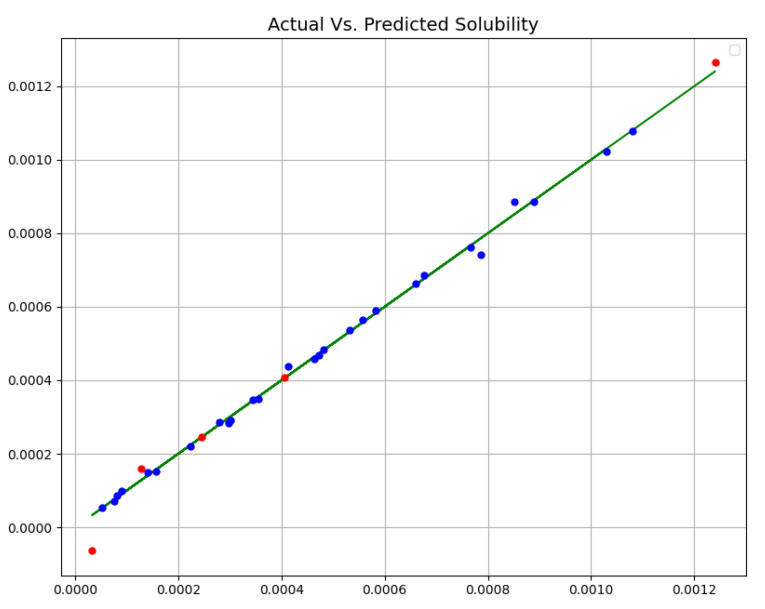
Real Vs. Forestalled Solubility (mole fraction) (GPR).

**Figure 4:**
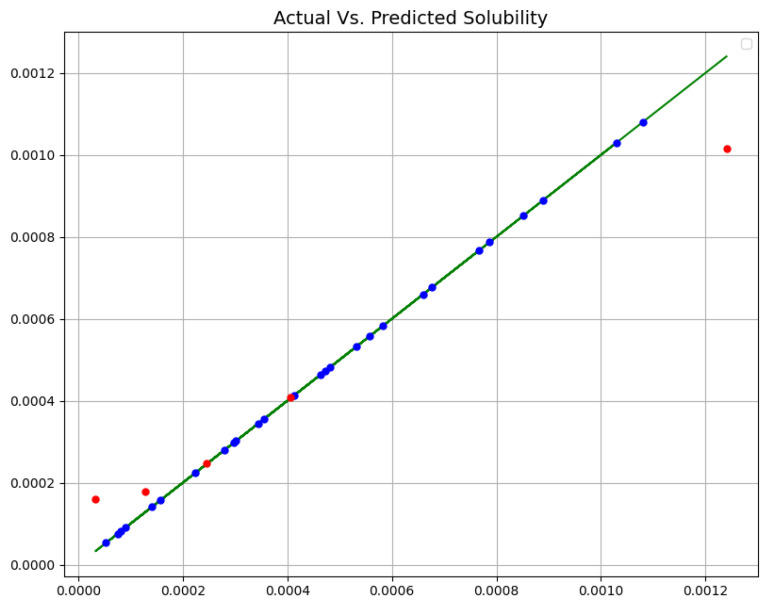
Real Vs. Forestalled Solubility (mole fraction) (KNN).

Table 2 enlists the final outcomes of all developed predictive models. Additionally, Figure 2, Figure 3 and Figure 4 schematically compare actual (experimental) and predicted (model-based) values via three proposed models (MLP, GPR, and KNN). In all diagrams, blue dots show forestalled amount, red dots show forestalled amount in trial, and the green line shows the real amount. According to this table and figures, we chose the GPR model as the most accurate model among these three models. Although the KNN model also shows close and accurate results, it can be seen in the test data that two points have a distance from the real values, and it has more MAPE.

**Figure 5:**
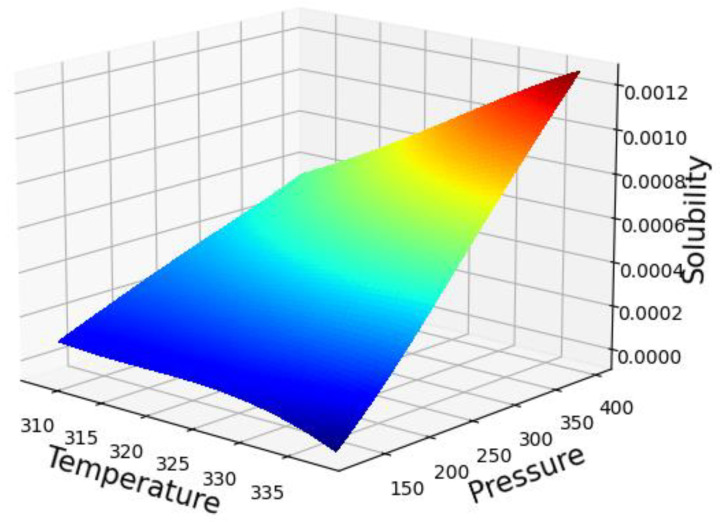
3D projection with GPR Model (pressure, bar/temperature, K/solubility, mole fraction).

**Figure 6:**
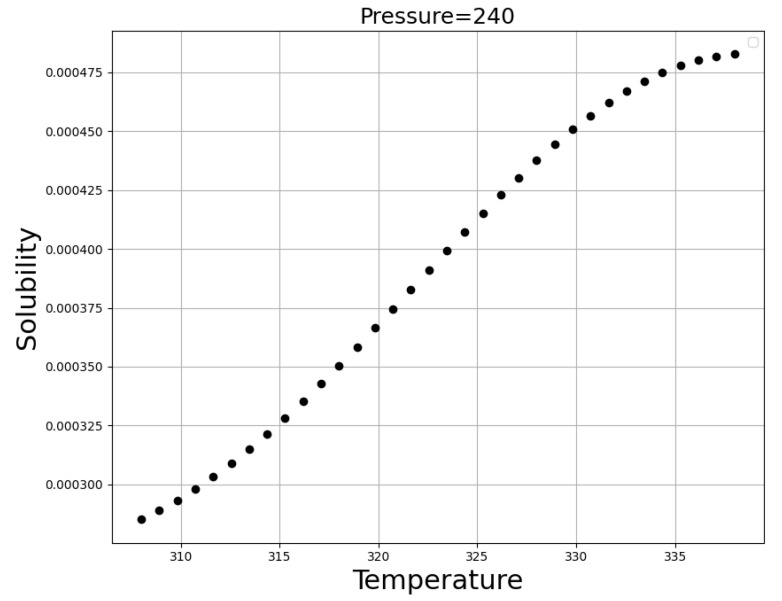
Trends for Temperature (K).

**Figure 7:**
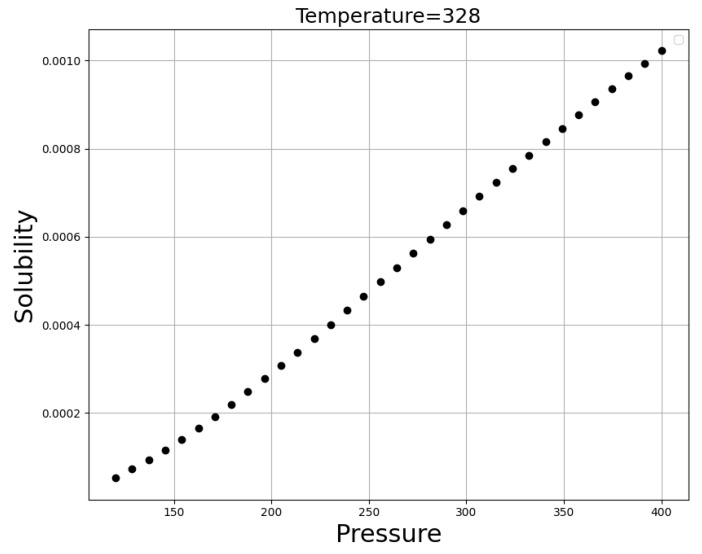
Trends for Pressure (bar).

The simultaneous influence of input values (temperature and pressure) on the solubility of oxaprozin is shown in Figure 5. If one of those parameters (temperature or pressure) keep constant, by changing the other one, two-dimensional Figure 6 and Figure 7, have been provided can illustrate this fact. The optimized parameters are illustrated in Table 3.

It is clear from Figure 6 and Figure 7 that an increase in the pressure causes a significant enhancement in the solubility value of a drug, which can be attributed to the increase of the molecular compression of solvent and improvement in the solubilizing power of SCCO_2_ [47,48,49]. Figure 7 demonstrates approximately five times improvement in the solubility of drugs by increasing the pressure from 120 to 410 bar. Regarding temperature, it can be said that this parameter has the opposite effect on the two competing parameters. An increase of temperature decreases the density of SCCO_2_, while increasing the sublimation pressure. Therefore, true analysis of these two parameters at the pressures below and above the cross-over pressure seems to be vital. At pressures below the cross-over pressure, the impact of density reduction prevails over the positive role of sublimation pressure and thus, an increase in temperature is equal to solubility reduction in SCCO_2_ fluid. By increasing the pressure to values higher than the cross-over pressure, the positive role of sublimation pressure prevails over the destructive role of density reduction and thus, an increase in the temperature considerably increases the oxaprozin solubility in the supercritical solvent. As presented in Table 3, the optimum values of pressure and temperature to gain the greatest value of solubility are predicted to be 400 bar and 338 K, respectively.

## 5. Conclusions

This paper was prominently focused on the prediction of oxaprozin solubility in SCCO_2_ fluid. To do this, machine learning (ML) techniques were employed to develop mathematical modeling and simulations to predict and optimize drug solubility. To make models on the small dataset, three learning methods were chosen: MLP, KNN, and GPR. There are 32 data points in the dataset, each with two input parameters (temperature and pressure) and one output parameter (solubility). Standard metrics were used to test the optimized models. Using the MSE metric, MLP, GPR, and KNN have error rates of 2.079 × 10^−8^, 2.173 × 10^−9^, and 1.372 × 10^−8^, respectively. In addition, they have R-squared scores of 0.868, 0.997, and 0.999, respectively. The optimal inputs are identical to the maximum possible values, and the output is a solubility of 1.26 × 10^−3^.

## Figures and Tables

**Table 1:** The dataset based on input and output.

No.	Temperature (K)	Pressure (bar)	Solubility (Mole Fraction)
1	3.08 × 10^2^	1.20 × 10^2^	8.19 × 10^−5^
2	3.08 × 10^2^	1.60 × 10^2^	1.58 × 10^−4^
3	3.08 × 10^2^	2.00 × 10^2^	2.24 × 10^−4^
4	3.08 × 10^2^	2.40 × 10^2^	2.80 × 10^−4^
5	3.08 × 10^2^	2.80 × 10^2^	3.44 × 10^−4^
6	3.08 × 10^2^	3.20 × 10^2^	4.06 × 10^−4^
7	3.08 × 10^2^	3.60 × 10^2^	4.73 × 10^−4^
8	3.08 × 10^2^	4.00 × 10^2^	5.33 × 10^−4^
9	3.18 × 10^2^	1.20 × 10^2^	7.55 × 10^−5^
10	3.18 × 10^2^	1.60 × 10^2^	1.41 × 10^−4^
11	3.18 × 10^2^	2.00 × 10^2^	2.45 × 10^−4^
12	3.18 × 10^2^	2.40 × 10^2^	3.56 × 10^−4^
13	3.18 × 10^2^	2.80 × 10^2^	4.64 × 10^−4^
14	3.18 × 10^2^	3.20 × 10^2^	5.58 × 10^−4^
15	3.18 × 10^2^	3.60 × 10^2^	6.60E × 10^−4^
16	3.18 × 10^2^	4.00 × 10^2^	7.66 × 10^−4^
17	3.28 × 10^2^	1.20 × 10^2^	5.34 × 10^−4^
18	3.28 × 10^2^	1.60 × 10^2^	1.28 × 10^−4^
19	3.28 × 10^2^	2.00 × 10^2^	3.02 × 10^−4^
20	3.28 × 10^2^	2.40 × 10^2^	4.14 × 10^−4^
21	3.28 × 10^2^	2.80 × 10^2^	5.82 × 10^−4^
22	3.28 × 10^2^	3.20 × 10^2^	7.87 × 10^−4^
23	3.28 × 10^2^	3.60 × 10^2^	8.51 × 10^−4^
24	3.28 × 10^2^	4.00 × 10^2^	1.03 × 10^−3^
25	3.38 × 10^2^	1.20 × 10^2^	3.3 × 10^−5^
26	3.38 × 10^2^	1.60 × 10^2^	9.09 × 10^−5^
27	3.38 × 10^2^	2.00 × 10^2^	2.98 × 10^−4^
28	3.38 × 10^2^	2.40 × 10^2^	4.81 × 10^−4^
29	3.38 × 10^2^	2.80 × 10^2^	6.77 × 10^−4^
30	3.38 × 10^2^	3.20 × 10^2^	8.89 × 10^−4^
31	3.38 × 10^2^	3.60 × 10^2^	1.08 × 10^−3^
32	3.38 × 10^2^	4.00 × 10^2^	1.24 × 10^−3^

**Table 2:** Outputs based on R^2^ and MSE.

Models	R^2^	MSE
MLP	0.868	2.079 × 10^−08^
GPR	0.997	2.173 × 10^−09^
KNN	0.999	1.372 × 10^−08^

**Table 3:** Optimized amount of inputs and output.

Temperature (K)	Pressure (bar)	Solubility
3.38 × 10^2^	4.00 × 10^2^	1.26 × 10^−3^

## Data Availability

All data are available within the published paper.

## Sample Availability

Samples of the compounds are available from the authors.
